# Supplementary material for: Patterns of Intron Gain and Loss in Fungi
Source: PLoS Biol. 2004 Nov 30;2(12):e422. doi: 10.1371/journal.pbio.0020422 (PMC532390; doi:10.1371/journal.pbio.0020422)
Supplement: Table S1 — Also available at http://genes.mit.edu/NielsenEtAl/. (4.3 MB ZIP). [file pbio.0020422.st001.zip › NielsenEtAl/html/53.html]

AN4099.1.NCU01089.1.MG08846.1.FG09059.1


```
 CLUSTAL W (1.82) Multiple Sequence Alignments - Introns Inserted


Sequence 1: NCU01089.1	589 aa
Sequence 2: FG09059.1	680 aa
Sequence 3: AN4099.1	787 aa
Sequence 4: MG08846.1	522 aa
Alignment Length: 879 aa
Number Identitical Residues: 173 aa
Alignment Score (without introns) 9872


MG08846.1 	------------------------------------------------------------
NCU01089.1	------------------------------------------------------------
FG09059.1 	------------------------------------------------------------
AN4099.1  	MVSTRHHPDDFPPPTSKSSSPLSPTSEPSGASSKTWLHTPTAILTLWLLISCPLVLWDAG
          	  ::   ... ...::.:::. :.::..:.:::.:   :.::  :     :..    .:.

MG08846.1 	------------------------------------------------------------
NCU01089.1	------------------------------------------------------------
FG09059.1 	------------------------------------------------------------
AN4099.1  	YCLLRPHSMPGGKYHSFWSGYKWYGTVDYIYGWPAFNANNGFTNAQAVLNLLETGGYLYY
          	 .   . : ....  :  :. .  .: .   . .: .:... :.:.:  .  .:..    

MG08846.1 	----------------------------------------------------------~-
NCU01089.1	----------------------------------------------------------~-
FG09059.1 	---MPEYGQPETRSEMVSKTYEIPMTDAKTNRGILALHLKYGWNTKFSQSNIDETKKK0K
AN4099.1  	LWVVYRHGTVAGDSKGSRGSQGTLSWLLATDKVVAGRMGATALLAAFSSSVSTVSKTV~F
          	       .     :.    :         :.    .     .  :  :.:    :..   

MG08846.1 	-~-------------------------------~--~-----------------------
NCU01089.1	-~-------------------------------~--~--------------------MAG
FG09059.1 	N~VKRMGMRRDRFRADHLRVG---------LWH~GL0IFMSQTYNTDIEHDSAALMAQNG
AN4099.1  	T0LTISGLQEYFSNFENIGHNEFWPMTAWIILN2GL~WIVVPVWNLHVLGEEIVSLLANG
          	.  .  .        .    ..  . ::      .         .     ..       .

MG08846.1 	-------~--------~--------MTSSKGPKKVVIIGAGAAG~--M~SCAATLAQHPD
NCU01089.1	E------~--------~------------ERKKKVLIVGAGAAG1--M~SCAYHLSNHPE
FG09059.1 	ETK----~--------~----------ANDGRKRVLIVGAGAAG2SSM~STAHHLSQHPD
AN4099.1  	TGAPRQI1RYPSITEW2QLQREYLQTMATSNRKRIAVVGAGAAG~--M0SCATTLSKHPA
          	   . .    .: :.  . . .  .  :..  *:: ::******   * * *  *::** 

MG08846.1 	KFKVTVLERSDVPGGQATTIPIDNQKFGAGWLNNGVQGGST0IFKHTFNFFKRYKHEPRE
NCU01089.1	KFDVTVIEAANYCGGQAFSIPLDKQKHGASWLNQGVQGGSY~IFHHTMTMFARQGYAANP
FG09059.1 	KFDITLIDAVDYCGGQAFSIPIDKERHGASWCNQGVQGGSY~IFHHTVTMFNRQGYHADP
AN4099.1  	KFDITLIDSVSQTGGQATSLPLDSSKYGASWLNDGVQGGSS~IFKHTFKFFQDYGYSPRE
          	**.:*:::  .  **** ::*:*..:.**.* *:******  **:**..:*    : .  

MG08846.1 	VKLQVSFGKGTEGFWTNCFPSKLVEELSSDIKKFGWFLKLVKYTMPVLGLVPIHVMMRLM
NCU01089.1	VKLQVSFGKG-DRFWTNVYPTKLLERHQSEVKRFYRMLSFLRWFELIFALLPIKYLMKLF
FG09059.1 	CELQVSFGKE-ETFWNNVFPTELLVRHQKEVRRLTTLLKVMRWFEIFFALLPLRLVFKLF
AN4099.1  	VKLQVAFGKGPDSFFTNVFPSPLVDRFQPEIKKLGRVLKVIKYGLPVLGILPVKVILKLF
          	 :***:*** .: *:.* :*: *: . . :::::  .*..:::   .:.::*:: :::*:

MG08846.1 	FFSKDFEHKMVYPLIALFLGTGNQTRHVPSAIVERLFDDPNMKLWD-FDKDTLLPNLPTM
NCU01089.1	WFSYEFANTVALPMVALFLGTGNYTPDVPTIILERLCTSPTYGMWYPADKESIASNMPPM
FG09059.1 	MFSEEFTNTIALPMTALFLGTGNETPRVPAMMFERLCTSPTYGMWYPADKNTVISNKPPM
AN4099.1  	RFSTEFGNRMLLPLLALFLGTGNQTPNVSCALLERLFQDPGMRLWE-YDSEGLMTNLPRM
          	 ** :* : :  *: ******** *  *.  :.***  .*   :*   *.: : .* * *

MG08846.1 	VTFDNLSEFYKDWMKDLVS-KGVDIRLGHEVVRVASRSHDGVVIES--RAAGSDGETP--
NCU01089.1	IVFPNFSNFYEDWHKDLVK-RGVNIRLSTEVTQILSRDKNGVVVKTIKRTPMPDNHNPTS
FG09059.1 	IVFPKFSEFYETWRKDLIS-RGVTVRLSTELTEIVQRNKHGVVVKLKPRTPMPDHHNP--
AN4099.1  	YTFPQLGMFYAQWKDDLEGNKGVRMLLGTEVE-IMHRAGDAVVLAL--HTP--DGKK---
          	 .* ::. **  * .**  .:** : *. *:  :  *  ..**:    ::.  * ..   

MG08846.1 	-----------------THEVFDDIVFCTL~ADDTLRLLGKTATRREKFVLSGAKFYDDI
NCU01089.1	AWATEDPVMNADADAEETTEHYDEIVICCL2ADTAKRLLGRTASLRERQVLGSAKFSDDI
FG09059.1 	--------AGGDPNAPVGEEKYDELVLCCL~ADTSKRVLGKTATWKEKKVLGSAKFSDDI
AN4099.1  	------------------TEAFDYMVLCTP~ADEALKLLGRHATWKERWVLGGVKFFDDV
          	                   * :* :*:*   ** : ::**: *: :*: **...** **:

MG08846.1 	TITHHDHAYFKKHYETTFQDDLCAEPKS----------KAQEEQVAFSTGK---DPKSPA
NCU01089.1	TITHHDSSYMRKHYENFFNPSQAVNSLSG---------VDHSSRVKFARDN---------
FG09059.1 	TITHNDAEYMKKHYENFYRDDLAVANVNG---------TDQSSRLNYAKTE---------
AN4099.1  	TVTHSDADYFNSIFETRIRDELVSTATGPSTTLSATTVSKRQDQLAFANAPSKCEEDGWA
          	*:** *  *:.. :*.  . .      . ::: ::::   :..:: ::   :... .. :

MG08846.1 	GFRPMYYTKSYKKDPTKIEMSFDCSNYQYQLLDAN--GKADDAGYEHVYQSIFLDKRNQ-
NCU01089.1	-FRPMYYIKPYDADLTKLEMCFDCTNYQAQFPPEV-------DFDNHVFQTIYLNKQRDG
FG09059.1 	-YRPMYYIKMYPEDKSKLEMCFDCTNYQSQFPEKV-------PFEQHVFQTIYLNKDRDG
AN4099.1  	GFNPMYYTHSYANEQDKIEMGFDCTNYQHQFRDAVGVGNSPYPAGQHVYQTIFLDKTRQ-
          	.:.**** : *  :  *:** ***:*** *:    . ..:     :**:*:*:*:* .: 

MG08846.1 	NLWTWNEIDESKIIKKQWWHQLGHRWQHYLRVVPGMMFLNG-RNKTWFAGSWTLV0NMHE
NCU01089.1	HLWTDDEIDESKIIRKDWWHQLCHSWTHYAFVVPWMWLLQG-RRHTRFASSWTMI~NAHE
FG09059.1 	KLWSDDEIDKDKIIRKDWWHQLCHSYTHYLFVVPWMMFLNA-KNHTRFAASWTLV~NAHE
AN4099.1  	DLWTWDSIDEDKVIGKKWWHQFGHRWQHYARVVPGMMFVNSLKNRTLFAGAWTMV0NMHE
          	.**: :.**:.*:* *.****: * : **  *** * :::. :.:* **.:**:: * **

MG08846.1 	LSCVSGIAAAYRLGADY---RKFDDFAENFFSKYLMLSHGVVYSME~EKRRKKAAPKS--
NCU01089.1	VAVMSGIAAAVDLGAQYPEDLERDGFALLSFRLYYLLIYGRWYRRK0DYGLKLNSSSPFY
FG09059.1 	VAVMSGIAAAVDLGATYPEDLENDKFAFLCFRLYYLLTYGKWYRRN~FTSKKYIKEHGET
AN4099.1  	IACISGIAAAYRLGASY---EHFDDFAQEVFAKYLFVCHGVWYKGE~GKKRA--------
          	:: :******  *** *    . * **   *  * :: :*  *  :              

MG08846.1 	---------------------------------------------------
NCU01089.1	FYNPAGKDKVMEYLVGGNCSMPEHLLQLLVDLGARLHIRNGEGHTPLVRCL
FG09059.1 	QAAMDGKSWATG-LYGSVYKGPG--VSEIERSCWREEIKQGASTGNLS---
AN4099.1  	---------------------------------------------------
          	
```
